# Supplementary material for: Divergence of wastewater SARS-CoV-2 and reported laboratory-confirmed COVID-19 incident case data coincident with wide-spread availability of at-home COVID-19 antigen tests
Source: PeerJ. 2023 Jun 27;11:e15631. doi: 10.7717/peerj.15631 (PMC10312197; doi:10.7717/peerj.15631)
Supplement: Supplemental Information 1 [file peerj-11-15631-s001.pdf]

Supporting Information

for

Divergence of wastewater SARS-CoV-2 and reported laboratory-confirmed COVID-19 incident case data coincident with wide-spread availability of at-home COVID-19 antigen tests

Alexandria B. Boehm[1]*, Marlene K. Wolfe[2], Bradley J. White[3], Bridgette Hughes[3], Dorothea Duong[3]

1. Department of Civil & Environmental Engineering, School of Engineering and Doerr School of Sustainability, Stanford University, 473 Via Ortega, Stanford, CA, USA 94305
2. Gangarosa Department of Environmental Health, Rollins School of Public Health, Emory University, 1518 Clifton Rd, Atlanta, GA, USA, 30322
3. Verily Life Sciences LLC, South San Francisco, CA, USA, 94080

* Author to whom correspondence should be addressed. Email: aboehm@stanford.edu, Tel: 650-724-9128

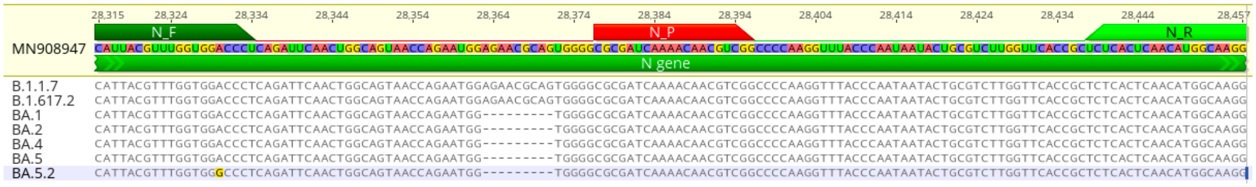

Figure S1. Location of the N gene assay primers (forward primer labeled N_F, reverse primer labeled N_R) and probe (labeled (N_P) relative to the genome of isolate MN908947. Sequences of representative variants circulating over the duration of this study are provided below the sequence of the MN908947 isolate and primer and probe locations. B.1.1.7 is Alpha, B.1.617.2 is Delta, BA.X are various Omicron sublineages. The sequence of BA.5.2 is highlighted at the bottom where there is one basepair mismatch, highlighted in yellow between the BA.5.2 consensus sequence and the forward primer. Less than 50% of BA.5* had this mutation. We tested whether this mutation affected quantification using synthetic gene blocks and found that its presence did not affect the performance of the assay and therefore do not expect this single SNP to have affected N gene quantification in samples containing BA.5.2 genomes.

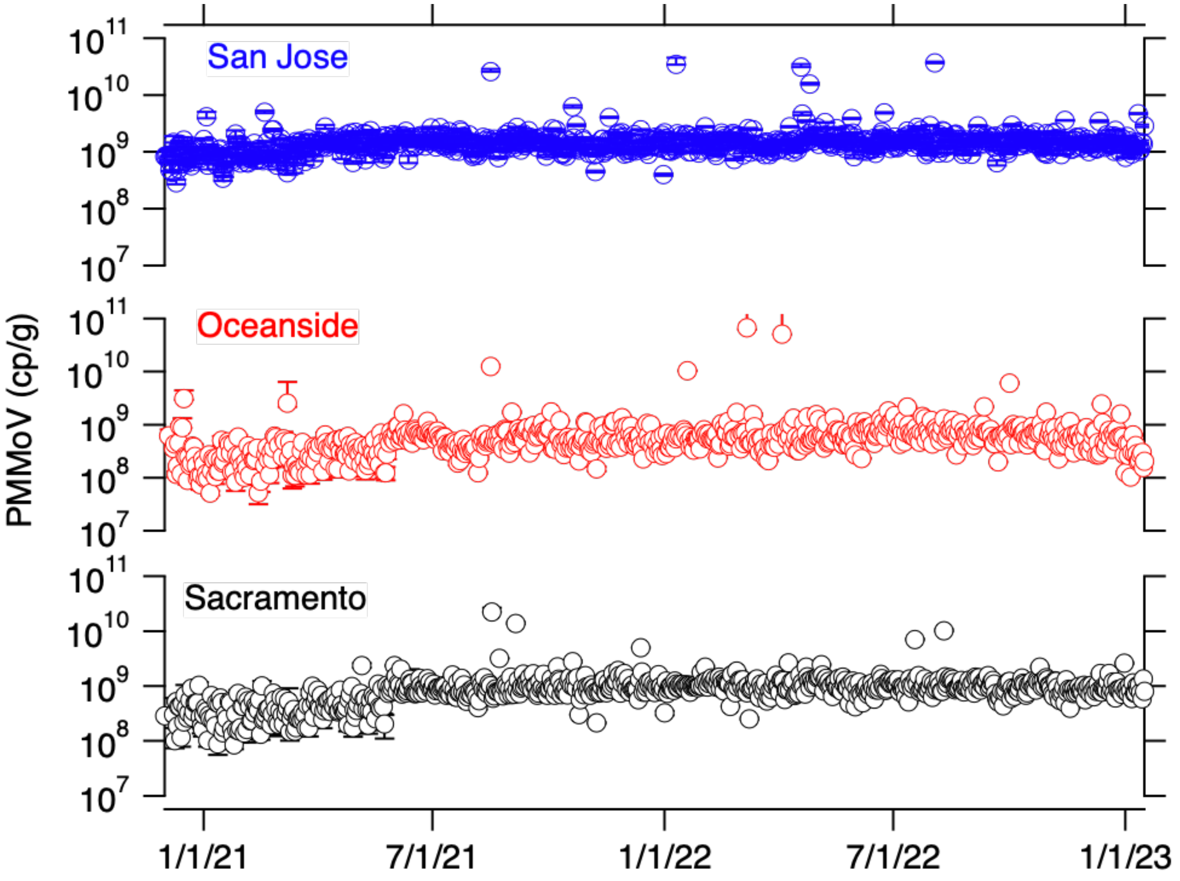

Figure S2. PMMoV concentrations in units of copies per gram dry weight and associated errors as represented by standard deviations. Errors are difficult to see in some cases because the size of the error bars are smaller than the symbol.

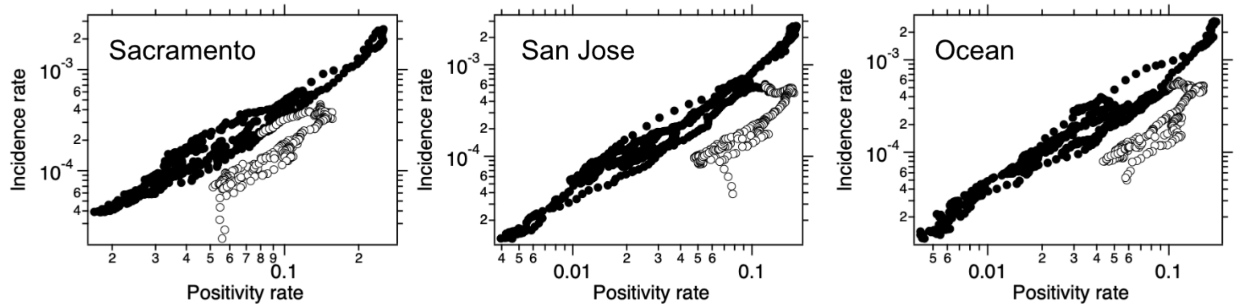

Figure S3. Relationship between incidence rate and positivity rate for the three POTWs. Here incidence rate and positivity rate are reported as fractions and the rates are the county-aggregated rates. Symbols colored black indicate data collected before 1 May 2022 and symbols colored white indicate data collected on or after 1 May 2022.

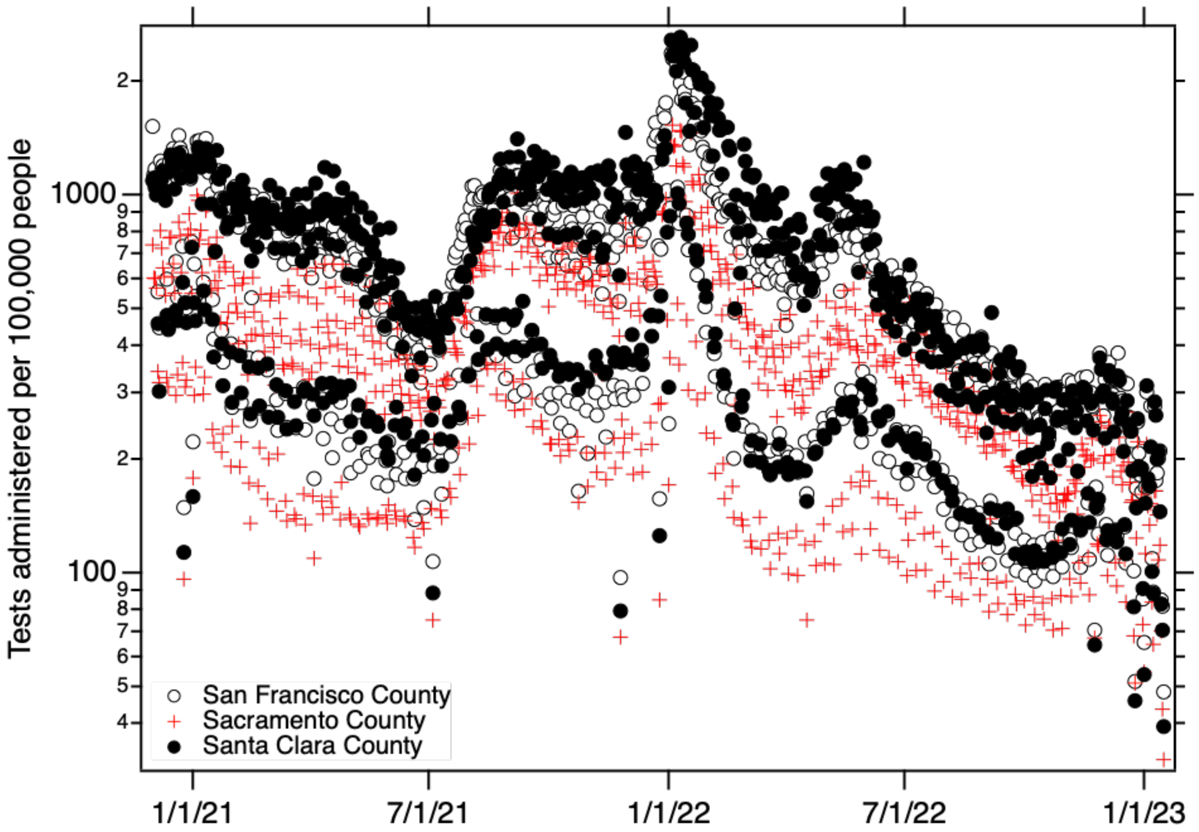

Figure S4. PCR tests administered per 100,000 people each day in the three counties. The lowest values are the tests administered on weekends and holidays.

Table S1. Coefficients for the model Z = b + m*X + n*D + k*(D*X) presented as equation 2 in the text where Z is $\log_{10}$-transformed positivity rate and Y is $\log_{10}$-transformed incidence rate. Adjusted R-square values and p-values for the model are provided, as well as the F statistic (and the degrees of freedom, DF). Coefficient values with standard errors are reported as well as p-values in parentheses. With Bonferroni corrections, p must be less than 0.0013 for alpha = 0.05. If n is significantly different from 0, then the intercept for the linear relationship between X and Y before and after 1 May 2022 is different and equal to b + n. If k is significantly different from 0, then the slope for the linear relationship between X and Y is different before and after 1 May 2022 and equal to m + k.

POTW	b	m	n	k	Adjusted R2	F-Statistic
SJ	-1.83±0.02 (0)	1.27±0.01 (0)	-0.32±0.06 ($<10^{-7}$)	0.19±0.05 (0.00016)	0.92 (10^{-15})	3188 on 3 and 773 DF
OS	-2.21±0.02 (0)	1.09±0.01 (0)	-0.64±0.11 ($<10^{-8}$)	-0.17 ±0.09 (0.07)	0.90 ($<10^{-15}$)	2360 on 3 and 733 DF
SAC	-1.91±0.02 (0)	1.43±0.02 (0)	-0.15±0.05 (0.0015)	0.23±0.04 ($<10^{-6}$)	0.93 ($<10^{-15}$)	3548 on 3 and 765 DF

Table S2. Coefficients for the model Y = b + m*X + n*D + k*(D*X) presented as equation 1 in the text where X is $\log_{10}$-transformed N/PMMoV (raw data) and Y is $\log_{10}$-transformed incidence rate (raw data). Adjusted R-square values and p-values for the model are provided, as well as the F statistic (and the degrees of freedom, DF). Coefficient values with standard errors are reported as well as p-values in parentheses. With Bonferroni corrections, p must be less than 0.0013 for alpha = 0.05. If n is significantly different from 0, then the intercept for the linear relationship between X and Y before and after 1 May 2022 is different and equal to b + n. If k is significantly different from 0, then the slope for the linear relationship between X and Y is different before and after 1 May 2022 and equal to m + k.

POTW	b	m	n	k	Adjusted R^2	F-statistic
SJ	-0.35±0.10 (3.1×10^{-4})	0.82±0.02 ($<10^{-100}$)	-1.5±0.22 ($<10^{-10}$)	-0.30±0.06 (1.3×10^{-6})	0.67 (10^{-15})	532.7 on 3 and 773 DF
OS	-1.9±0.08 ($<10^{-86}$)	0.49±0.02 ($<10^{-98}$)	-2.3±0.32 ($<10^{-12}$)	-0.59 ±0.09 ($<10^{-9}$)	0.45 ($<10^{-15}$)	205.9 on 3 and 733 DF
SAC	-0.77±0.09 ($<10^{-16}$)	0.75±0.02 ($<10^{-100}$)	-0.77±0.21 (2.1×10^{-4})	-0.13±0.06 (0.03)	0.63 ($<10^{-15}$)	428 on 3 and 765 DF
